# The Importance of Vaccination, Variants and Time Point of SARS-CoV-2 Infection in Pregnancy for Stillbirth and Preterm Birth Risk: An Analysis of the CRONOS Register Study

**DOI:** 10.3390/jcm13061522

**Published:** 2024-03-07

**Authors:** Antonella Iannaccone, Alexandra Gellhaus, Beatrix Reisch, Mark Dzietko, Boerge Schmidt, Laven Mavarani, Katrina Kraft, Kristin Andresen, Rainer Kimmig, Ulrich Pecks, Ekkehard Schleußner

**Affiliations:** 1 Department of Obstetrics and Gynecology, University Hospital, University of Duisburg-Essen, 45147 Essen, Germany; alexandra.gellhaus@uk-essen.de (A.G.); beatrix.reisch@uk-essen.de (B.R.); rainer.kimmig@uk-essen.de (R.K.); 2 Department of Pediatrics, University Hospital Essen, University of Duisburg-Essen, 45147 Essen, Germany; mark.dzietko@uk-essen.de; 3 Institute for Medical Informatics, Biometry and Epidemiology, University of Duisburg-Essen, 45147 Essen, Germany; boerge.schmidt@uk-essen.de (B.S.);; 4 Department of Obstetrics and Gynecology, University Hospital of Lübeck, 23562 Lübeck, Germany; katrina.kraft@uksh.de; 5 Department of Obstetrics and Gynecology, University Hospital of Kiel, 24105 Kiel, Germany; kristin.andresen@uksh.de; 6 Department of Obstetrics and Gynecology, University Hospital of Würzburg, 97080 Würzburg, Germany; pecks_u@ukw.de; 7 Department of Obstetrics, Jena University Hospital, 07747 Jena, Germany; ekkehard.schleussner@med.uni-jena.de

**Keywords:** vaccination, preterm birth, stillbirth, COVID -19, SARS-CoV-2, alpha, delta, omicron, wild-type

## Abstract

**Background:** The risk of preterm birth (PTB) and stillbirth increases after a SARS-CoV-2 infection during gestation. We aimed to estimate the risk depending on gestational age at infection (early <28 + 0 and late ≥28 weeks of gestation, WoG), virus variants, severity of infection, and vaccination. **Methods:** PTB was divided into early PTB (<32 + 0) and late PTB (32 + 0–36 + 6 WoG). The prospective register COVID-19 Related Obstetrics and Neonatal Outcome Study (CRONOS) included 8032 pregnant women with a confirmed SARS-CoV-2 infection from 3 April 2020 to 31 December 2022, in Germany and Austria. **Results:** Stillbirth and early preterm births rates were higher during the Alpha (1.56% and 3.13%) and Delta (1.56% and 3.44%) waves than during the Omicron wave (0.53% and 1.39%). Early SARS-CoV-2 infection increased the risk for stillbirth (aRR 5.76, 95% CI 3.07–10.83) and early PTB before 32 + 0 (aRR, 6.07, 95% CI 3.65–10.09). Hospital admission increased the risks further, especially in the case of ICU admission. Vaccination against SARS-CoV-2 significantly reduced the risk of stillbirth (aRR 0.32, 95% CI 0.16–0.83). **Conclusions:** This multicentric prospective study shows an increased risk of stillbirth and preterm birth after infection early in pregnancy and therefore the importance of obstetrical surveillance thereafter. Vaccination offers effective protection.

## 1. Introduction

Since the outbreak of the SARS-CoV-2 (severe acute respiratory syndrome coronavirus 2) pandemic in 2020, questions about the risk of infection during pregnancy have arisen. Overall, the pandemic situation had a tremendously negative impact on maternal and neonatal health [1].

Pregnancy is a physiological state that predisposes women to respiratory complications from viral infection [2,3].

SARS-CoV and MERS-CoV (*Middle East respiratory syndrome-related coronavirus*) were responsible for severe complications during pregnancy [4]. SARS-CoV-2 belongs to the same β-coronavirus subgroup, and it has a genome similarity of about 80% and 50% with SARS-CoV and MERS-CoV, respectively [5].

COVID-19 (coronavirus disease 2019), the disease induced by the SARS-CoV-2 infection, caused more severe respiratory complications in pregnant than in non-pregnant women, with a two to three times higher risk of being admitted to an intensive care unit, needing invasive ventilation and extracorporeal membrane oxygenation (ECMO) or even of dying [6]. The risk increases with ongoing pregnancy and is highest at around 32 weeks of gestation [7], most probably due to physiological changes in pregnancy leading to restricted pulmonary residual capacity and alterations in immune defense [8].

Several studies showed that risk of adverse perinatal outcome increases after maternal SARS-CoV-2 infection [9,10].

The gestational week at the time of infection appears to be an important determining factor [11].

In particular, studies have shown that the risk of preterm birth (PTB) and stillbirth increased after an infection in the first half of pregnancy [12], and that the severity of the disease does not play an important role [11]. Yet, hospitalization seems to be important in determining the respiratory [10] but also obstetrical [12] risks. 

Stillbirth definitions vary between countries around the globe and differ especially between high- and low-income countries around the world [13]. The American College of Obstetricians and Gynecologists (ACOG) defines stillbirth as delivery of fetus which shows no signs of life, e.g., absence of breathing, heart beats, pulsations in umbilical cord are absent, no voluntary movement of muscle. The suggested requirement is to report fetal deaths at 20 weeks or more of gestation (if the gestational age is known) or a weight greater than or equal to 350 g, if the gestational age is not known. The cut-off of 350 g is the 50th percentile for weight at 20 weeks’ gestation [14]. Regarding preterm births (PTB), these are those that occur at under 37 weeks’ gestational age; however, the low-gestational age cutoff, or that used to distinguish preterm birth from spontaneous abortion, varies by location [15]. PTB is further subdivided on the basis of gestational age (GA): extremely preterm (<28 weeks), very preterm (28–<32 weeks) and moderate or late preterm (32–<37 completed weeks of gestation) [16]. Preterm births account for 75% of perinatal mortality and more than half the long-term morbidity [15].

Different virus variants circulated in Germany and epidemiological data on variant dominance are available from the German Surveillance System of the Robert Koch Institute (https://edoc.rki.de/bitstream/handle/176904/9483/EB-10-2022-Phaseneinteilung.pdf, accessed on 30 January 2024). Recent data suggest an increased rate of stillbirth during the Delta period [17]. PTB rates were significantly lower during the Omicron period [18]. An epidemiological study revealed recently that the risk of preterm birth is lower when the vaccination rate is higher [19].

The main aim of this study is to compare the PTB and stillbirth risk depending on the time point of infection, dominant virus variant, severity of disease requiring hospitalization, and vaccination status.

## 2. Materials and Methods

### 2.1. Study Design and Setting

From 3 April 2020 to 31 December 2022, the data of 8032 women with acute or previous SARS-CoV-2 infection (positive PCR or antigen test) at any time during their pregnancy and who, regardless of indication, were cared for in one of the participating obstetric departments were collected in the multicentric and prospective register COVID-19 Related Obstetrics and Neonatal Outcome Study (CRONOS). CRONOS is an observational study established by the German Society of Perinatal Medicine (DGPM) to rapidly provide data to counsel women with SARS-CoV-2-infection during pregnancy. Information on the study is available at www.dgpm-online.org and the German Clinical Trials Register (DRKS00021208). All German maternity hospitals were invited to participate in the study. Obstetrical Units covering almost 1/3 of the deliveries in Germany as well as the Kepler University Hospital in Linz, Austria, participated and included cases. Methodology and study results were published recently [7,20,21,22,23,24,25]. Approval of the Institutional Ethics Board was obtained for the study (University Hospital Schleswig-Holstein in Kiel, file number D 451/20) and for each study site separately.

### 2.2. Data Capture and Study Variables

After obtaining informed consent from patients, information on the demographic characteristics, medical and obstetrical history, infection-specific symptoms and/or treatments, obstetrical and neonatal outcomes of the current pregnancy were entered by each participating hospital in the cloud-based electronic data capture platform of the service provider castoredc.com (Amsterdam, The Netherlands). The virus variant was calculated based on information and epidemiological data on variant dominance from the German Surveillance System of the Robert Koch Institute (https://edoc.rki.de/bitstream/handle/176904/9483/EB-10-2022-Phaseneinteilung.pdf, accessed on 30 January 2024). From the first recorded cases (27 January 2020) until the end of the second wave on the 28th of February 2021, the wild-type virus circulated in Germany. During the third wave, from the 1 March 2021 until the end of the summer plateau on the 1 August 2021, the variant of concern (VOC) was Alpha. From the 2nd of August, the new VOC Delta took over, causing the fourth wave until the 26 December 2021. From the 27th of December 2021 the VOC was Omicron.

#### Endpoints

The primary endpoints were stillbirth after 20 weeks of gestation WoG and PTB (livebirth before completed 37 WoG). Those were further classified as early PTB (<32 + 0 (WoG)) or late PTB (from 32 + 0) and. The timing of exposure was dichotomized into early (<28 + 0 weeks of gestation) and late (third trimester ≥ 28 weeks) infections.

### 2.3. Statistical Analysis

To evaluate the impact of the time point of infection in pregnancy, of vaccination against SARS-CoV-2, and of dominant virus variant on the endpoints, we conducted a stepwise statistical analysis strategy.

1. A descriptive analysis of baseline characteristics of the population: categorical variables are presented as absolute and relative frequencies (*N*/%); continuous baseline variables were shown as mean and standard deviation (mean ± SD) for each group (Table 1).
2. A descriptive bivariate analysis to assess the proportion of stillbirth, early (<32 + 0), and late (32 + 0–36 + 6) PTB, depending on the time point of infection, virus variant, and vaccination status (Table 2).
3. Finally, multivariate log-binomial models for calculating adjusted relative risk (aRR) and corresponding 95% confidence intervals (95% CI) were used to correct the risk for maternal characteristics and common risk factors.

Statistical analyses were performed using Statistical Package for Social Sciences (SPSS; version 29; IBM Corp, Armonk, NY, USA) for Windows (Microsoft, Redmond, WA, USA). Missed values are dropped by default in SPSS for log-binomial models; therefore, sample sizes may differ in the statistical analyses. Inferential statistics were used in a descriptive manner. Thus, no significance levels were determined, and no adjustment for multiplicity was applied. However, *p* values < 0.05 were considered to be statistically significant.

## 3. Results

### 3.1. Descriptive Statistics

The baseline maternal demographic and clinical characteristics of the 8032 infected pregnant women are presented in Table 1.

The mean maternal age was 31.11 ± 5.35 years and the mean gestational week at delivery was 38.15 ± 3.31.

The mean BMI was 29.06 ± 5.83 kg/m^2^. The study population included 3% twin pregnancies and 4.1% pregnancies were induced by medically assisted reproduction. In 1.7% of women, a history of preterm birth was indicated.

Of the pregnant women, 4.3% smoked during the pregnancy.

Some 71.9% of the women were inpatients at the time of the infection, with 10.7% admitted for symptoms related to COVID-19 and 48.35% for obstetrics complication or delivery. Of the patients, 2.9% were admitted to the intensive care units (ICU).

Most women were infected with SARS-CoV-2 during the Omicron dominant phase (3390, 42.2%). During the 3 months of Alpha as VOC, the registered number of infections was 766 (9.5%). During the period of 5 months when Delta was prevalent, there were 1542 (19.2%) recorded infections during pregnancy. During the first 13 months of the pandemic, 2334 cases of infection were reported in which only the wild-type virus was detected.

In the population studied, 26.8% women were vaccinated against SARS-CoV-2 at the time of infection.

Of the women analyzed, 3119 (38.8%) acquired the infection early in pregnancy and 4913 (61.2%) in the third trimester.

### 3.2. Rate of Stillbirth, Early and Late PTB

Seventy patients (0.87%) suffered a stillbirth after 20 + 0 week of gestation (WoG), but rates vary depending on the time point of infection in pregnancy, virus variant, and vaccination status (Table 2).

Depending on the time point of infection, the stillbirth rate was higher after an early infection compared with an infection in the third trimester: 1.47% (46/70) versus 0.49% (24/70).

The rate of stillbirth was higher during the Alpha and Delta periods: 1.56% in both periods (12/766 and 24/1542), almost three times higher than during the wild-type and Omicron periods: 0.69% (16/2334) and 0.53% (18/3390, Table 2).

Further, in the vaccinated women, the stillbirth rate was almost half compared to the unvaccinated group: 0.51% (11/2156) versus 1.04% (54/5174).

Eight hundred and thirty-five pregnant women (10.39%) had a preterm birth: 165 (2.05%) deliveries were early preterm and 670 (8.43%) occurred after 32 + 0 WoG (Table 2)

In the case of early infection, the rate of PTB before 32 + 0 WoG was higher: 3.63% (113/3119) versus 1.05% (52/4913) after a late infection. In cases of late infection, the rate of late PTB was higher: 9.32% (458/4913) compared to 6.80% (212/3119) after early infection (Table 2).

Regarding the virus types, the rate of early PTB was higher among pregnant women infected during the Alpha and Delta period: 3.13% (24/766) and 3.44% (53/1542) versus 1.76% (41/2334) and 1.39% (47/3390) during the wild-type and Omicron periods. The rate of late PTB was lowest during the Omicron period with 7.35% (249/3390). During the wild-type, Alpha, and Delta waves, the rates of late PTB were 9.30% (217/2334), 9.40% (72/766), and 8.56% (132/1542) (Table 2).

In cases where vaccinations had been given, the rate of early PTB almost halved: 2.66% (138/5174) in the unvaccinated population versus 1.25% (27/2156) among vaccinated pregnant individuals. Also, the rate of late PTB was lower in the vaccinated population: 6.54% (141/2156) versus 9.43% (488/5147) in the unvaccinated population.

### 3.3. Adjusted Relative Risks of Stillbirth and Preterm Delivery

Having any symptoms, independent of the severity, reduced the risk of stillbirth (adjusted relative risk—aRR 0.16, 95% CI 0.26–1.125) and early PTB (aRR 0.35, 95% CI 0.20–0.61), but had no influence on the aRR for late PTB (aRR 0.99, 95% CI 0.77–1.27, Table 3). Inpatients admitted for symptoms related to the SARS-CoV-2 infection, had increased risks, with an aRR of 2.86 for stillbirth (95% CI 1.08–8.02) and 1.49 for early PTB (95% CI 1.14–5.47). The aRR for late PTB was 1.71 in the case of symptomatic disease with hospital admission (95% CI 1.25–2.51). Hospital admission for obstetrical reasons increased the aRR for stillbirth by 5.50 times (95% CI 2.90–10.41), for early PTB 4.40 times (95% CI 2.80–6.90), and for late PTB 2.06 times (95% CI 1.70–2.51, Table 3).

The admission to an intensive care unit (ICU) increased the adjusted relative risk of stillbirth 3.44 times (95% CI 1.04–11.38), the adjusted relative risk of early PTB was 11.66 (95% CI 5.22–26.05), for late PTB 1.98 (95% CI 1.31–3.00, Table 3).

Being infected during the Alpha or Delta periods, compared with other variants, increased the adjusted relative risk of stillbirth (aRR 1.76, 95% CI 0.99–3.08) and early PTB (aRR 1.45, 95% CI 0.96–2.18). The risk of late PTB was not increased (aRR 0.89, 95% CI 0.72–1.10, Table 3).

Regarding the timepoint of infection, the relative risks of stillbirth and early PTB were higher after an infection early in pregnancy: aRR 5.76, 95% CI 3.07–10.83 and aRR 6.07, 95% CI 3.65–10.09. The risk of late PTB is not increased after an infection early in pregnancy (aRR 0.79, 95% CI 0.62–1.01, Table 3).

Being vaccinated against SARS-CoV 2 reduced the risk of stillbirth (aRR 0.32 CI 0.11–0.92, *p* = 0.035). The risks of early and late preterm birth were also lower: aRR 0.65 (95% CI 0.38–1.11) and aRR 0.73 (95% CI 0.58–0.92, Table 3).

## 4. Discussion

### 4.1. Principal Findings

The rate of early preterm delivery and stillbirth was higher after infection before the third trimester and during the Alpha and Delta periods of SARS-CoV-2 infections. The rate of stillbirth and early PTB were lower in the vaccinated population. SARS-CoV-2 infection early in pregnancy and infection during the Alpha and Delta periods increased the risk for stillbirth and early preterm birth. Having symptoms independent of the severity reduced the risk for PTB, especially for early PTB. Hospital admission for COVID-19-related symptoms as a surrogate of a more severe course of COVID-19 disease increased the risks. Vaccination against SARS-CoV-2 significantly reduced the risks, especially for stillbirth. It is known that the risk of stillbirth after a SARS-CoV-2 infection in pregnancy is increased [26]. A recent meta-analysis estimated that the odds ratio of stillbirth in COVID-19 compared to non-COVID-19 pregnant women was 1.89 [27]. In 2022, we reported the increased risk especially after early infection in a smaller cohort based on our CRONOS register data [12]. Since the rate and risk of stillbirth is dependent on gestational age, it is not possible to exclude that not only the infection, but gestational age itself affected our results. Pregnant individuals may have presented in hospital due to the diagnosis of intrauterine demise and have had an early infection detected through hospital screening. In a retrospective study from 2010, the stillbirth rate was higher early in gestation, but the risk increased late in gestation, especially at 42 weeks [28]. The hypothesis is that the infection produced an inflammation in the maternal body affecting the fetus correlated with an adverse outcome increasing the risk of stillbirth and preterm birth.

Pregnant women with COVID-19 versus without COVID-19 are more likely to deliver preterm and could have an increased risk of maternal death and of being admitted to the intensive care unit [29]. Pregnant women especially early in the second trimester seem to be more susceptibly and tend to have a worse outcome after infection in this period [18]. In 2022, Piekos et al. [11] analyzed the risk of maternal and pregnancy complications depending on the time point of infection in 882 infected pregnant women compared with 889 matched control pregnant women. An infection in the first and second trimester increased the risk of stillbirth and early PTB. They also found that gestational week at infection could predict the week of gestation at delivery, but the severity of the infection was not correlated with gestational age at delivery. In our first analysis of a smaller cohort from CRONOS, which included 1149 pregnant women [12], and in this study including 8032 infections in pregnancy, the admission to hospital as a surrogate for the severity of infection increased the risk for stillbirth and preterm birth. In the case of admission to an ICU, the risk increase was even higher. Differently applied statistical models, but also the population size, could be explanations for the different results, since the number of infected women was lower in the cohort of Piekos and colleagues [11].

The prevalence of congenital malformations in SARS-CoV-2-infected women did not increase in the CRONOS register compared to the known European prevalence [30]. In countries where the Zika Virus is endemic, the clinical differentiation between the two entities with similar symptoms is difficult and represents an issue due to the late diagnosis of congenital Zika syndrome, which includes microcephaly and neurodevelopmental delay [31].

The Delta and the Alpha variants showed increased transmissibility shortly after being discovered compared to earlier variants. It soon became clear that the Delta variant was not only more transmissible but potentially more severe, leading to higher rates of hospitalization and complications in pregnant women, resulting in an increase in adverse maternal and fetal outcomes in affected pregnancies [32].

The study of Favre [17] examined the period before and after Delta. In this analysis, the higher risk for maternal health but also the increased risk for obstetrical complications especially preterm birth and stillbirth during Delta is evident. During the Delta period, the stillbirth rate was 2.8%, higher than the one observed in our study. The reason for this could be the smaller examined population with n = 1402, n = 262, and n = 391 for pre-Delta, Delta, and post-Delta, respectively.

Depending on different virus variants, different PTB rates were described at an academic center in New York (US) [18]: 9.9% during Omicron compared with a peak in PTB rate of 20.3% during the original period, which is of course much higher than the 11.06% PTB rate registered during the wild-type period in our study. The German preterm birth rate is 9%, stable in recent years [33] whereas the PTB birth rate in the US is higher, at 10.38% in 2022, with a higher rate among black non-Hispanic women (14.59%) [34]. The reason in this case could also be the different size of the studied populations, since in the study, 8983 women were analyzed but only 638 were infected.

Regarding vaccination, the main body of research evaluates the safety of vaccination against SARS-CoV-2 in pregnancy [35,36]. The role of vaccination and its effect on obstetrical outcomes has not yet been intensively examined. One recent study showed that the risk increase for PTB after infection was lower when the vaccination rate in the population was higher [19]. Our data support not only the safety of vaccination against SARS-CoV-2 in pregnancy, they underline the importance of vaccination by lowering the risk for adverse outcomes like stillbirth and preterm birth in the vaccinated population.

### 4.2. Clinical Implications

Our data provide a valid instrument for consulting pregnant women on the modulation of risk after SARS-CoV-2 infection during pregnancy and the role of vaccination. Overall, obstetrical complications seemed to be lower during the Omicron wave. However, an infection early in pregnancy has been shown to increase the risks of the analyzed obstetrical complications. Therefore, intensive obstetrical surveillance after an early pregnancy infection is still mandatory. Vaccination offers effective protection against the risk of stillbirth and preterm birth (PTB), and women should be encouraged to receive immunization against SARS-CoV-2 infection. Vaccination during pregnancy is a delicate topic that arouses a lot of skepticism in patients. The pandemic is a case in point. Since the results of the vaccination studies, including pregnant individuals, were available later than those regarding other populations, immunization in pregnancy started later. An earlier analysis of this important group could have prevented many complications making it a current and exemplary issue, an important lesson for the future.

### 4.3. Research Implications

The reasons for the susceptibility of the studied pregnancy complications after an early infection need further evaluation. The placenta may play a crucial role. Different molecular pathways could be modulated at this stage, potentially contributing to the elevated risk of placental dysfunction, which correlates with the increased risk of medically induced preterm birth. We also hypothesize that infections in the late second trimester could trigger an escalation of inflammation, possibly accounting for most cases of spontaneous preterm birth. Given the multifactorial nature of preterm birth and the often unexplained reasons for stillbirth, confirming this hypothesis will be challenging. Detailed and extensive biomolecular placental research could provide answers, but clinical examination and matching cases with a control group may also yield valuable insights.

### 4.4. Strengths and Limitations

We present one of the largest cohorts of infected women from high-income countries such as Germany and Austria, offering detailed insights into their symptoms, pregnancy outcomes, and medical histories. Our study meticulously examines the impact of vaccination, reaffirming its crucial role in preventing the elevated risks of stillbirth and early preterm birth. The recruited infected pregnant women primarily originated from obstetrical hospitals, predominantly tertiary care centers. As a result, most cases involved hospitalized and often high-risk individuals. We acknowledge that future studies should include an uninfected group for a comprehensive comparison. Additionally, we did not differentiate between spontaneous and medically indicated preterm deliveries, recognizing that both may be influenced by the infection. In the case of stillbirth, we did not categorize by specific causes, considering any delivery involving an unviable fetus as a stillbirth.

## 5. Conclusions

Our analysis reaffirms the critical need for obstetric surveillance following a SARS-CoV-2 infection in early pregnancy. The heightened risks of preterm birth and stillbirth are substantiated in this study. The presence of mild symptoms appears to have little relevance, while severe symptomatic cases necessitating treatment and hospitalization emerge as significant risk factors for preterm birth and stillbirth. Moreover, this analysis provides further support for the presumed protective effect of vaccination, particularly in preventing stillbirth.

## Figures and Tables

**Table 1:** Baseline and pregnancy characteristics of the study participants.

	Mean/*N*	SD/%	Missing
Maternal age (years)	31.1	5.4	37
BMI at inclusion (kg/m^2^)	29.1	5.8	2282
Week of gestation at birth	38.2	3.3	735
PTB	835	11.4	735
History of PTB	138	1.7%	17
IVF	292	4.1%	959
Multiple pregnancy	237	3%	76
Smoking during pregnancy	333	4.1%	240
Any symptoms	6025	81.6%	644
Vaccine against SARS-CoV-2	2156	26.8%	702
Hospital admission	5585	71.9%	265
Hospital admission for COVID-19	828	10.7%	264
Hospital admission for obstetric reasons	3756	48.3%	264
ICU admission	223	2.9%	267

**Table 2:** Descriptive bivariate analysis of stillbirth, early preterm and late preterm birth depending on time point of infection, virus variant and vaccination status.

			Stillbirth (*N* = 70, 0.87%)		Early PTB (*N* = 165, 2.05%)		Late PTB (*N* = 670, 8.34%)	
	*N*	%	*N*	%	*N*	%	*N*	%
**Early infection**	3119	38.8%	46	1.47%	113	3.62%	212	6.80%
**Late infection**	4913	61.2%	24	0.49%	52	1.05%	458	9.32%
**Wild-type**	2334	29.1%	16	0.69%	41	1.76%	217	9.30%
**Alpha**	766	9.5%	12	1.56%	24	3.13%	72	9.40%
**Delta**	1542	19.2%	24	1.56%	53	3.44%	132	8.56%
**Omicron**	3390	42.2%	18	0.53%	47	1.39%	249	7.35%
**Vaccine**	2156	26.8%	11	0.51%	27	1.25%	141	6.54%
**No vaccine**	5174	64.4%	54	1.04%	138	2.66%	488	9.43%

**Table 3:** Multivariate log-bin model regarding the adjusted relative risk (aRR) of stillbirth, early and late PTB depending on symptomatic infection, hospital admission due to SARS-CoV 2 infection, for obstetrical reasons, ICU admission, being infected with Alpha or Delta variants compared to other virus types, early infection compared to late infection and being vaccinated. Significant *p*-values are marked in bold. The 95% lower and upper confidence intervals (CI) are indicated. RR adjusted for maternal age, BMI, multiples and history of miscarriage/preterm delivery.

Outcome	Parameter	Symptomatic	CoV Admission	Ob Admission	ICU Admission	Alpha Delta vs. Others	Early vs. Late	Vaccine
**Stillbirth**	aRR	0.16	**2.86**	**5.50**	**3.44**	1.76	**5.76**	**0.32**
	95% CI	0.26–1.25	**1.08–8.02**	**2.90–10.1**	**1.04–11.38**	0.99–3.08	**3.07–10.83**	**0.16–0.83**
	*p*-Value	0.573	**0.035**	**<0.001**	**0.043**	0.055	**<0.001**	**0.019**
**Early PTB**	aRR	**0.35**	**2.49**	**4.40**	**11.66**	1.45	**6.07**	0.65
	95% CI	**0.20–0.61**	**1.14–4.47**	**2.80–6.91**	**5.22–26.05**	0.96–2.18	**3.65–10.09**	0.38–1.11
	*p*-Value	**<0.001**	**0.023**	**<0.001**	**<0.001**	0.077	**<0.001**	0.112
**Late PTB**	aRR	0.99	**1.71**	**2.06**	**1.98**	0.89	0.79	**0.73**
	95% CI	0.77–1.27	**1.25–2.51**	**1.70–2.51**	**1.31–3.00**	0.72–1.10	0.62–1.01	**0.58–0.92**
	*p*-Value	0.924	**<0.001**	**<0.001**	**<0.001**	0.269	0.065	**0.008**

## Data Availability

Data are contained within the article.
